# Clinical Diagnosis and Management of Fetal Alcohol Spectrum Disorder and Sensory Processing Disorder in Children

**DOI:** 10.3390/children11010108

**Published:** 2024-01-16

**Authors:** Lorel Breuer, Jacob R. Greenmyer, Ted Wilson

**Affiliations:** 1 Department of Biology, Winona State University, Winona, MN 55987, USA; lorel.breuer@go.winona.edu; 2 Pediatric Hematology and Oncology, Mayo Clinic, Rochester, MN 55905, USA; greenmyer.jacob@mayo.edu

**Keywords:** fetal alcohol spectrum disorder (FASD), sensory processing disorders, facial dysmorphology, alcohol related neurodevelopmental disorder, prenatal alcohol exposure

## Abstract

Fetal alcohol spectrum disorder (FASD) is commonly misdiagnosed because of the complexity of presentation and multiple diagnostic criteria. FASD includes four categorical entities (fetal alcohol syndrome, partial fetal alcohol syndrome, alcohol related neurodevelopmental disorder, and alcohol related birth defects). The four FASD diagnostic criteria are facial dysmorphology, growth deficiency, central nervous system dysfunction, and prenatal alcohol exposure. Sensory processing disorders (SPDs) are common in FASD and are observed as inappropriate behavioral responses to environmental stimuli. These can be either a sensory-based motor disorder, sensory discrimination disorder, or sensory modulation disorder. A child with SPD may experience challenges with their fine motor coordination, gross motor coordination, organizational challenges, or behavioral regulation impairments. FASD requires a multidimensional approach to intervention. Although FASD cannot be cured, symptoms can be managed with sleep-based therapies, sensory integration, and cognitive therapies. This paper reviews SPDs in FASD and the interventions that can be used by practitioners to help improve their therapeutic management, although it is unlikely that any single intervention will be the right choice for all patients.

## 1. Introduction

Fetal alcohol spectrum disorder (FASD) refers to a continuum of adverse outcomes resulting from prenatal alcohol exposure (PAE). The four categorical entities are: fetal alcohol syndrome (FAS); partial fetal alcohol syndrome (PFAS); alcohol related neurodevelopmental disorder (ARND); and alcohol related birth defects (ARBDs) [1]. Clinical features of the FASD phenotype can include facial dysmorphology, growth deficiency, and central nervous system dysfunction. FAS is the most severe and distinct subset of FASD and includes all aspects of the clinical phenotype [2]. FASD is a common neurodevelopmental disorder with a high prevalence of comorbid conditions including sensory processing disorders (SPDs) [3]. FASD is prevalent in about 5% of school aged children [4].

The neurocognitive features of FASD are thought to be attributable to altered neuronal synaptic connectivity [3,5]. Sensory processing disorders (SPDs) represent a common comorbidity of FASD that are manifested as inappropriate behavioral reactions to environmental stimuli. SPDs impair an individual’s ability to respond appropriately to his or her surroundings [6]. SPDs have three broad subtypes: sensory-based motor disorder, sensory discrimination disorder, and sensory modulation disorder [7].

Clinical intervention for SPD is in part determined by symptom severity, which can be managed by a variety of interventions. Interventions to assist children with FASD, SPD, or both, emphasize non-pharmaceutical interventions such as sensory integration, counseling (for both child and family), hands-on activities, sleep-based therapies, introduction of structure and routines, and cognitive control therapies. Pharmaceutical interventions are also often required. These pharmaceutical interventions often target a wide range of co-occurring disorders but may not be unique to FASD or SPDs specifically [8,9]. Since SPDs are commonly comorbid with FASDs, early recognition, diagnosis, and referral for SPDs is an important aspect of the care for children and families with FASD. The implementation of early intervention measures aims to enhance outcomes, including the advancement of activities replicating daily living skills and the mitigation of potential secondary conditions such as academic underachievement, deficiencies in social skills, and overall performance in various age-appropriate environments. Management and interventions can be applied in diverse settings including homes, schools, and the community [10].

This paper examines the different subtypes of FASDs and discusses how both FASDs and SPDs negatively affect a child’s daily functioning with emphasis on SPDs in clinical treatments which provide management strategies needed for understanding which clinical therapies are possible and needed.

## 2. Biochemical Mechanisms That Influence FASD

The brain has a long developmental time window leading to many opportunities for prenatal alcohol exposure over the course of the 38-week gestation. Exposure to alcohol can disrupt the interactions between cells, resulting in impairments in the central nervous system that could lead to FASD [2]. Cell adhesion molecules (CAMs) play a critical role in the development of the pathways that create our nervous system. Prenatal alcohol exposure, especially in the first trimester of pregnancy, can disrupt neuronal proliferation, migration, and synapse formation made possible by cell-adhesion molecules [2,11]. Alcohols have a specific binding site on the L1 CAM that may influence their ability to fix neurons, axons, and dendrites in their proper locations, leading to FASD [12]. Furthermore, alcohol can also influence the kinases that influence the CAMs functions [13]. These CAM-mediated activities are vital for the growth of the embryo, fetus, and newborn, resulting in developmental and behavioral abnormalities after birth, and could play a role in the development of FASD [11,14].

Human prenatal alcohol exposure is also known to be associated with epigenetic changes in DNA methylation leading to alterations in gene expression in the child [15]. A recent study of gene expression in the cerebral cortex of mice following gestational alcohol exposure is suggestive of the complicated nature of how alcohol exposure can affect the newborn; after 60 days postnatally, 765 genes were differentially upregulated and 597 were downregulated in a sex-specific manner [16]. Blood serum from children with FASD appears to have lower concentrations of NGF and BDNF and higher concentrations of TNF- α and IL-1α relative to healthy controls, suggestive of differences in neuronal development, neuronal plasticity, and inflammatory processes [17]. Our understanding of how PAE influences gene expression is exceedingly complicated; the potential of epigenetic changes in one human generation to be expressed in following generations makes it very difficult to provide simple answers.

## 3. Diagnostic Criteria of Fetal Alcohol Spectrum Disorders

### 3.1. Fetal Alcohol Spectrum Disorders Diagnostic Criteria

FASD is a permanent birth defect and is not curable but is completely preventable. The diagnosis of FASD involves a comprehensive assessment of various factors across four categories [1]. FASD can be diagnosed by facial dysmorphology, growth deficiency, central nervous system dysfunction, and a history of PAE [2]. FASD is often misdiagnosed due to comorbidities and varying clinical phenotypes. Clinics often do not have the staff volume or specialized expertise necessary to diagnose and treat the large number of people who have FASD. FASD is diagnosed by healthcare providers based on the presence or absence of criteria [18].

### 3.2. Prenatal Alcohol Exposure

The first criterion for a FASD diagnosis is PAE, which occurs when an embryo or fetus is exposed to alcohol from a woman drinking alcohol while pregnant, either deliberately or inadvertently. PAE can cause damage to the brain and central nervous system, which can lead to hyperactivity, impulsivity, and a lack of self-control [19]. The four FASD diagnostic criteria are caused by alcohol, leading to alterations in development and the volume of a child’s brain. Alcohol can have a postnatal effect on a child’s senses, motor coordination, cognition, and social and emotional processing. Confirmation of PAE is necessary for all FASD diagnoses except for FAS. Confirmation can be obtained through methods using surveys, interviews, questionnaires, clinical observation, self-reporting, or the detection of biomarkers in maternal and newborn blood, hair, breath, urine, and the placenta [20]. Most biomarkers indicate alcohol exposure within the past 24 h. Maternal breath alcohol concentrations can be used as a biomarker for the estimation of alcohol levels in both the mother and fetus [21]. Testing for phosphatidylethanol (PETH) can also be utilized to evaluate alcohol exposure at birth. PETH is a byproduct of alcohol intake and can be found in dried blood spots. A positive PETH test suggests alcohol consumption in the month leading up to birth as it is a long-term biomarker of alcohol use [22]. Finding appropriate biomarkers is challenging due to the expenses involved and availability of samples, despite the fact that biomarkers offer greater benefits and potentially higher reliability compared to self-reports [23]. Endorsement of a single uniform biomarker remains to be made by a professional medical organization [20].

### 3.3. Paternal Influences on FASD

Gestational exposure of the fetus to alcohol due to maternal alcohol exposure has traditionally been viewed as the most significant predictive factor for the development of FASD; however, pre-conception paternal exposure may also contribute to FASD. A human study of 164,151 couples with paternal alcohol consumption prior to conception revealed a significant increase in fetal birth defects, although FASD was not examined [24]. In a mouse model paternal alcohol exposure is associated with many of the same craniofacial deformities associated with FASD in humans [25]. In a mouse model of the exposure of sires to dietary alcohol for 70 days prior to, but not during, mating with non-ethanol-exposed female mice was evaluated. Paternal alcohol exposure was associated with a significant 10% increase in the duration of gestation [26]. In addition, paternal alcohol exposure also led to a significant body weight reduction in 1-week-old male and female pups of 25% and 15%, respectively [26]. In a mouse model, paternal alcohol exposure has been shown to lead to epigenetic changes that could even potentially cause intergenerational changes in hormonal and genetic expression [27].

Given that single parent families are becoming ever more common in the USA and Western countries, maternal knowledge of prior paternal alcohol exposure may not always be readily available adding an additional layer of complication to interpreting FASD in a child and making management decisions. Therefore, it is probable that paternal alcohol exposure prior to conception can potentially even influence postnatal FASD across generations due to epigenetic influences, as mentioned previously. Future studies will be needed to more fully understand the significance of paternal alcohol exposure in FASD.

### 3.4. Facial Dysmorphology

**Figure 1:**
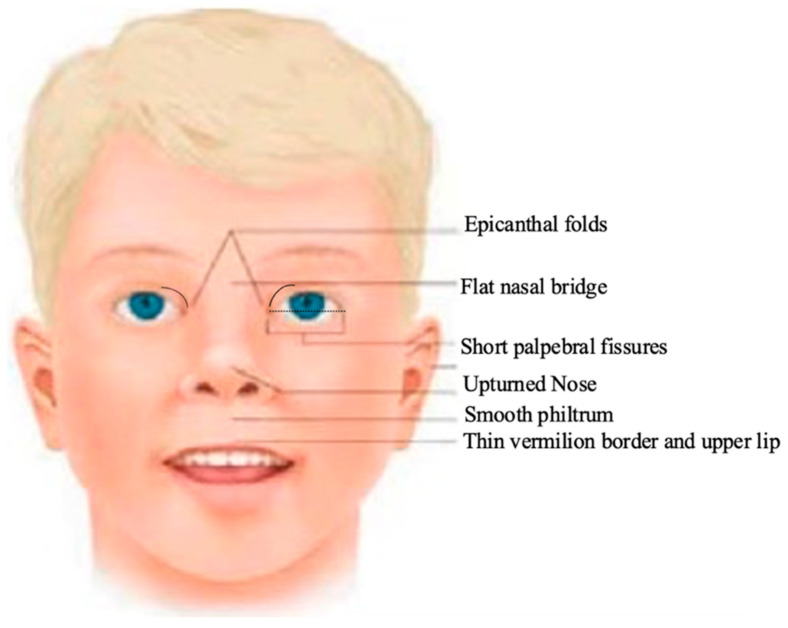
Facial dysmorphology characteristics that result from PAE [modified from [28]].

Common facial dysmorphic characteristics from FASDs include epicanthal folds, a flat nasal bridge, short palpebral fissures, an upturned nose, smooth philtrum, and a thin vermilion border on the upper lip (Figure 1). Facial malformations in children are often accompanied by impairments in vision and hearing, speech and language delays, and a wide range of impairments in emotional regulation [28].

### 3.5. Growth Deficiency

Physical development may be impacted by prenatal alcohol exposure, and postnatal adversity resulting in a growth impairment in height, weight, and head circumference. A growth deficiency is commonly identified when age–gender height and weight fall below the tenth percentile [14]. Growth delays increase a child’s overall health risks by affecting their general health and cognitive and physical development. Insufficient brain volume and structure size resulting from PAE lead to impaired cognitive function [29]. Research conducted on rats has experimentally confirmed that PAE exposure during fetal development can lead to a diminished weight of vital organs such as the brain, liver, and placenta [30,31].

### 3.6. Central Nervous System Dysfunctions in Fetal Alcohol Spectrum Disorders

Individuals with a FASD may struggle with challenges ranging from basic sensory processing to cognitive processing. They may have problems with the organogenesis of multiple organ systems including the heart, kidneys, bones, or morphogenic problems leading to impaired hearing or vision. Cognitive impairments are often associated with speech and language disabilities, motor disabilities, memory impairments, impaired cognitive reasoning, thinking, and functioning, as well as other learning disabilities. Many of these challenges interfere with day-to-day living and can lead to behavioral issues, or academic achievement issues [18].

## 4. Fetal Alcohol Disorders

### 4.1. Fetal Alcohol Syndrome

FAS is the most severe subtype within the spectrum of FASD. Children with FAS meet the diagnostic criteria in FASD. SPDs are common in FAS due to brain and central nervous system abnormalities. These children may struggle with hyperactivity, behavioral problems, learning and memory problems, social problems, facial abnormalities, and development problems [19]. They may also have difficulties with sleeping, eating, or performing other activities within daily living. The nurturing environment in which a child with FAS is raised, whether positive or negative, can influence their development and the long-term severity of their symptoms for the duration of their lives [32].

### 4.2. Partial Fetal Alcohol Syndrome

A diagnosis of PFAS requires fewer deficits in growth and facial features. The diagnosis of PFAS requires a confirmation of alcohol exposure, some facial dysmorphology, or components thereof, and often central nervous system dysfunction. Three of the four criteria must be confirmed for this diagnosis [33]. Children with PFAS experience neurobehavioral, and intellectual impairments [34].

### 4.3. Alcohol Related Neurodevelopmental Disorder

By far the most common manifestation of FASD is the categorical subtype of ARND which is about 85% of all FASD. ARND emphasizes the associated cognitive impairments, behavioral, or educational difficulties. These in turn provide the neuropsychological basis for an SPD. In this context, an SPD is the manifestation of multiple developmental incapacities that have an intricate impact on the neurological maturation and functioning of children [9].

### 4.4. Alcohol Related Birth Defects

Children with ARBD usually endure problems with their organs and bones as a result of alcohol exposure. However, they do not have neurodevelopmental deficits. PAE must be confirmed alongside at least one congenital defect affecting one of the organ systems, particularly the heart, bones, hearing, or kidneys. Typically, this co-exists as a secondary disability [19]. During the early stages of development, the risk of ARBD is elevated. Alcohol is not metabolized by the fetus because the fetus possesses an underdeveloped liver, hindering its ability to metabolize alcohol or the more toxic acetaldehyde created by maternal alcohol metabolism in the maternal liver at the same rate as the mother. Consequently, an accumulation of fetal acetaldehyde leads to reactive oxygen species resulting in the potential for DNA damage in the fetus [29]. Furthermore, when maternal alcohol appears in the fetus’ blood it can change the transportation of nutrients and blood flow through the placenta leading to fetal nutrient deficiencies [30].

## 5. Sensory Processing Disorders

### 5.1. Sensory Processing Disorder

How a child perceives and processes sensory information in their brain is disrupted by SPDs resulting in frequent inappropriate responses to sensory stimuli. A child with this condition may have several impairments ranging from motor coordination, including both fine and gross coordination, to behavioral issues, and challenges with organization [10].

### 5.2. Sensory Process Disorder Subtypes

**Figure 2:**
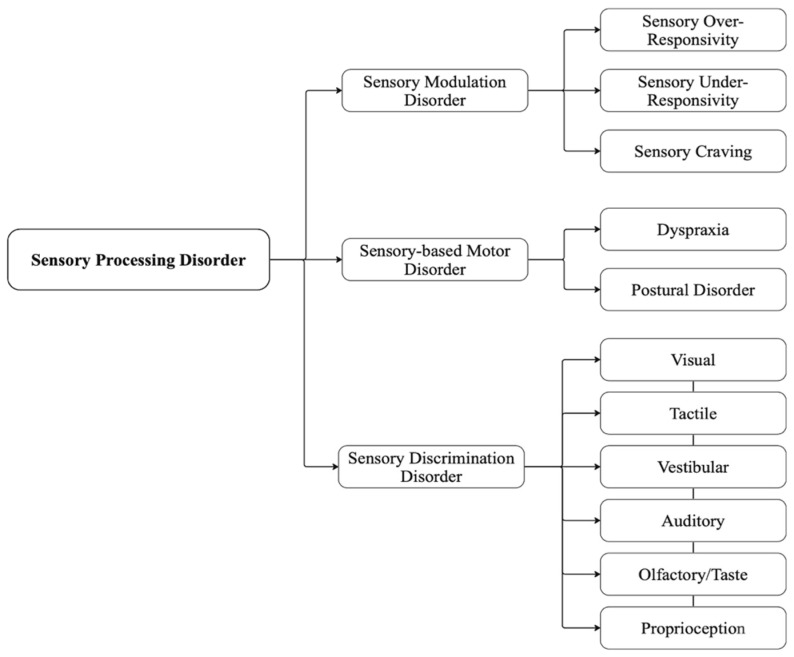
Characterization of the three primary sensory processing disorders. Adopted from [35].

Sensory modulation disorder, sensory-based motor disorder, and sensory discrimination disorder are the three subtypes of SPD (Figure 2). When a child reacts inappropriately to stimuli, they may overreact, underreact, or have sensory cravings [6]. These three types of responses make up the subtype of sensory modulation disorder. A child struggling with this condition could have trouble controlling their reactions to sensory information [34]. Dyspraxia, which impairs motor movements, and postural disorder, which affects equilibrium and bodily stability, are the two kinds of sensory-based motor disorder. Lastly, sensory discrimination disorder impairs the ability to determine between the input’s intensity and origin. All of the senses—visual, auditory, tactile, olfactory, taste, vestibular (balance and spatial orientation), and proprioceptive (body awareness)—are affected by this condition. Since SPD is a broad phenotype, the manifestation will vary greatly across individual children. The interventions for SPDs are tailored to the child’s specific needs and may differ across settings in the child’s life [7].

## 6. Correlative Relationship between Fetal Alcohol Spectrum Disorder and Sensory Processing Disorders

Sensory processing difficulties and behavioral issues are interrelated in children with an FASD. There is a higher likelihood of functional behavioral deficits in socialization, attention, rule-breaking, cognitive difficulties, and aggression in children with FASDs who also have SPD deficits. Deficits in auditory processing and sensory modulation may increase the prevalence of behavioral deficits due to their limited ability to adapt behaviorally [6]. On a global scale, the prevalence rate of FASDs is 0.77%. FAS has a prevalence rate of 0.15% and PAE related conditions have a prevalence rate of 0.77%. When individuals are exposed to higher levels of PAE, they are more likely to have SPDs. The perception of sensation can be significantly impacted by PAE. Further research is needed to fully understand the prevalence and intricacies of sensory processing symptoms [18,36]. Children with FASDs will process sensory information differently from those without the disorder and often have difficulty in managing their responses to sensory input which may vary widely across settings. They can have a strong desire for sensory stimulation but struggle to maintain focus on their surroundings [37]. They are easily distracted by new stimuli, which leads to increased hyperactivity. Their behavior can range from being highly attentive and inflexible to completely withdrawn and disengaged.

Children with FASDs can also be exceptionally sensitive to stimuli and may actively try to avoid environments with sensory overload. They struggle with habituation and can become overwhelmed by sensory input, causing exaggerated reactions and irritability [10]. Carr (2010) compared children with PAE to children with both PFAS and ARND, they found that those with PFAS or ARND had greater sensory processing deficit than those with PAE [34]. However, Jirikowic (2020) found that higher levels of reported PAE overall were correlated with sensory processing symptoms in a large percentage of children aged 3 to 11 years old compared to children of the lower levels of PAE, or unconfirmed PAE [36]. Exposing a fetus to alcohol on a regular basis or at higher doses with more harmful levels increases the likelihood of a child having a worse prognosis of FASD and SPD.

## 7. Therapeutic Management Considerations

### 7.1. Therapy

FASD clinics use a wide range of expertise; however, the number of children with these conditions exceeds the number of clinics with sufficient capacity for intervention [9]. Healthcare providers, such as occupational therapists, physical therapists, registered nurses, and pediatricians, support a child who has FASD and an SPD through various treatment regimens and interventions. The interventions are based on developmental trajectories the presence of current developmental delays and emphasize successful transition into adulthood [38]. Therapy and intervention plans can vary and are tailored specifically for helping the individual child [9].

### 7.2. Therapeutic Interventions

There are numerous approaches to therapeutic intervention including the use of sleep-based therapies, cognitive control therapy, introduction of structure and routines [8] sensory integration, counseling, medications, and other hands-on activities which can have a significant impact on a child’s disorder management. These therapies can assist individuals in the development of appropriate behavioral responses required for effectively daily functioning [9].

#### 7.2.1. Sleep-Based Therapies

Brain damage is a consequence of PAE that children with FASDs experience, resulting in sleep struggles. A strong correlation between abnormal sleep patterns and abnormalities in sensory processing has been demonstrated [3]. Children who have a hard time sleeping may benefit from working with their occupational therapist to develop interventions specific to sleep and self-regulation utilizing sensory-based therapies.

#### 7.2.2. Sensory Integration

A key concept in managing SPDs is the recognition that the observed behavioral manifestations are due to brain damage and are thus best conceptualized as impairments. This helps families, teachers, and others understand that the inappropriate behaviors seen in FASDs and SPDs are the result of brain damage not just willful acting out. This demonstrates the need for impairment-oriented interventions for SPDs that include sensorimotor manipulation and sensory-based approaches. These integrative approaches seek to improve activity performance and participation by focusing on the specific body structure and function that is impaired and attempting to correct the problem. Sensorimotor approaches can include interventions such as therapy balls and movement therapies. Sensory based approaches can include strategies like weighted vests or sound therapy [39]. Sensory integration is a technique that has been shown to be beneficial in children with SPDs. It can help a child’s cognitive and social abilities, as well as other deficiencies (Table 1). This may be particularly beneficial for children who have sensory oversensitivity [7].

#### 7.2.3. Cognitive Therapies

The performance-oriented category proposes another set of intervention strategies. Cognitive-based therapies aim to facilitate a child’s learning of an activity by teaching them how to use skill-specific strategies. These therapy strategies aim to improve performance for specific skills and participation by focusing on activity performance improvement. This can be accomplished through direct skill teaching or cognitive interventions. These have been suggested to be promising for children, particularly those with motor coordination issues [39]. Cognitive control therapy aims to change a child’s thought patterns through a series of steps, teaching them how to think and engage in self-regulation strategies. When used as part of an intervention for children with FASDs, it has been shown to improve behavior [40].

### 7.3. Therapy Intervention Benefits

Riley (2003) discovered that while there may be an improvement in behavior and adaptive functioning, meta-cognitive abilities and neuropsychological functioning were less positively impacted [41]. Effective therapeutic interventions in a variety of domains benefit children and adolescents with FASD. Ordenewitz (2021) suggests that self-regulation and social interaction skills training has a long-term beneficial effect on attention and behavior [42]. There is also evidence that parent-child sessions are a promising strategy. These findings show there are more benefits for the child when their family and surroundings adhere to their therapy and intervention plans or take classes to understand their child’s challenges.

### 7.4. Intervention Effectiveness

Intervention plans can be implemented during appointments in the clinic, as well as in the home, and at school. The environment can influence the management of a child’s condition [39]. Guardians, teachers, and other community members or specialists who adhere to the suggested therapy and interventions plans outside of the occupational therapist’s clinic will observe better outcomes for the child [8]. Early diagnosis, the effectiveness of the intervention, and adherence to the plans are a few factors that may significantly affect the child’s prognosis. A child’s potential risk of developing a secondary disease may be reduced by an early diagnosis. As the child deals with difficult situations in the real world, the effectiveness of an intervention becomes increasingly important. The number of high-quality therapies for managing FASDs is limited [40].

### 7.5. Complexity of Interventional Choices for the Practitioner

**Figure 3:**
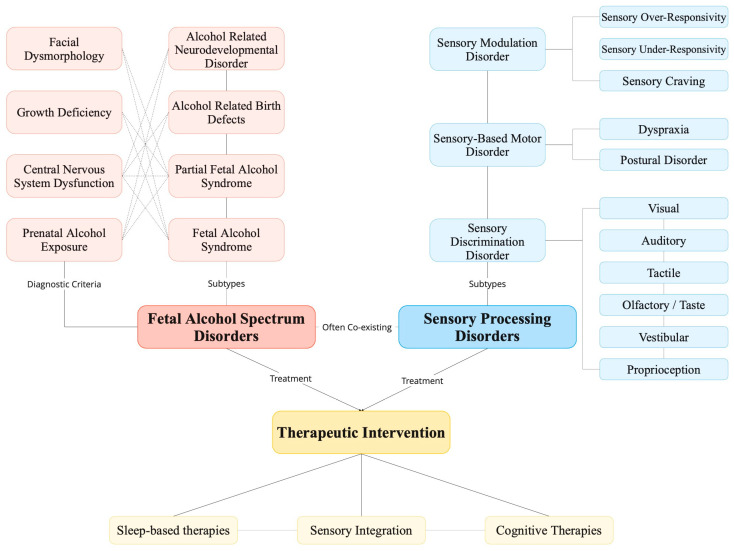
Four types of disorders make up the fetal alcohol spectrum disorders which commonly co-occur with sensory processing disorders. These conditions are not curable but can be managed with intervention strategies.

Multiple levels of healthcare provider are available to help children manage the symptoms of an FASD or SPD. Practitioners need to customize their strategies to incorporate various intervention methods such as sensory integration, medication, establishing routines, sleep-based therapies, counseling for both the child and guardian, and other techniques (Figure 3). Continued improvements in FASD diagnosis and therapeutic intervention will certainly improve future outcomes for the child, parent(s), and clinician. It is crucial for children to have a solid support system and receive the therapeutic interventions that provide the child with the opportunity to experience improved self-confidence and self-control.

## 8. Limitations

The diagnosis of SPD relies on observation rather than standardized tools, creating uncertainty about potential interventional risks. The financial and time burdens of SPDs can be significant for families, yet these aspects have not been thoroughly studied. Further research is needed to establish standardized assessment tools and a more consistent approach to diagnosing SPDs. Practitioners need to remember that the diverse causative nature of FASDs and SPDs makes it unlikely that a single therapeutic intervention will be the equally effective choice for all patients.

## 9. Summary

Fetal alcohol exposure is preventable and can have long-term effects on the child including risk disorders, such as FASDs and/or SPDs that frequently co-occur. The potential for paternal alcohol exposure, and even potential epigenetic changes from a prior generational exposure to influence FASD further complicates these conditions. A child with an SPD has trouble understanding and responding to the sensory stimuli that are presented to them in their daily lives. SPDs are not reversible/curable, are extremely complex, and expresses with a variety of symptoms. Sensory processing disorders come in the forms of sensory-based motor disorder, sensory modulation disorder, and sensory discrimination disorder. They can have an impact on a child’s behavior, cognitive thinking, attention, fine and gross motor coordination, as well as all their senses, including proprioception and vestibular. These may significantly affect a person’s daily life and quality of life if untreated. However, both FASDs and SPDs can be managed with the right interventions and therapies, and if the practitioner is knowledgeable about how to choose the right therapeutic intervention that meets the needs of the patient, and their family.

## Figures and Tables

**Table 1:** Intervention management considerations for fetal alcohol spectrum disorders and sensory processing disorders.

Intervention Method	Outcomes
Sleep-based Therapies	Improves sleeping
Sensory Integration	Improves cognition and social abilities. Especially useful for sensory oversensitivity
Cognitive Therapies	Improves performance, participation, and self-regulation. Especially useful for motor coordination
